# Peritoneal Tuberculosis in Western Countries: A Rare Case With Concurrent Helminthic Infection

**DOI:** 10.7759/cureus.54438

**Published:** 2024-02-19

**Authors:** Chai Wei Tong, Mina Sarofim, Ruwanthi Wijayawardana, David L Morris

**Affiliations:** 1 Surgery, St George Hospital, Kogarah, AUS; 2 School of Medicine, University of New South Wales, Sydney, AUS

**Keywords:** rare cause of acute abdominal pain, ascites of unexplained origin, peritoneal tuberculosis (tb), helminthic infection, abdominal tuberculosis

## Abstract

This case report presents a rare case of peritoneal tuberculosis (TB) coexisting with a helminthic infection in a 25-year-old female residing in Australia, highlighting the diagnostic challenges posed by abdominal TB. Despite the low incidence of TB in Western countries, abdominal TB remains a diagnostic dilemma due to its nonspecific symptoms and potential mimicry of other abdominal pathologies. The case highlights the importance of considering TB as a differential diagnosis of unexplained abdominal symptoms, particularly in individuals with a history of travel or previous residence in high-endemic regions. A multidisciplinary approach involving infectious disease specialists, radiologists, and surgeons is essential for comprehensive management. Prompt initiation of anti-TB therapy is recommended once diagnosis is confirmed.

## Introduction

Australia has one of the lowest tuberculosis (TB) rates in the world, with approximately 6.7 cases per 100,000 population. More than 90% of these cases are diagnosed in populations who were born or spent a significant amount of time in countries endemic for TB [1]. Abdominal TB accounts for <3% of all TB cases worldwide and is extremely rare in Western countries [2,3]. Symptoms and signs are often non-specific and mimic other more common abdominal pathologies making diagnosis difficult. Here we present a rare case of tuberculous peritonitis with a concurrent helminthic infection.

## Case presentation

We present a 25-year-old female who presented to the emergency department of a tertiary hospital with a six-day history of generalized abdominal pain, associated with anorexia and the presence of worms in her stool. Initially diagnosed with a helminthic infection, she was discharged with oral Mebendazole. However, her symptoms worsened, prompting a second presentation to the hospital with escalating abdominal pain and new fevers. There were no respiratory symptoms and she had no significant medical or surgical history. Although she was of Nepalese origin, she had been residing in Australia for the preceding two years and had not traveled overseas.

**Figure 1:**
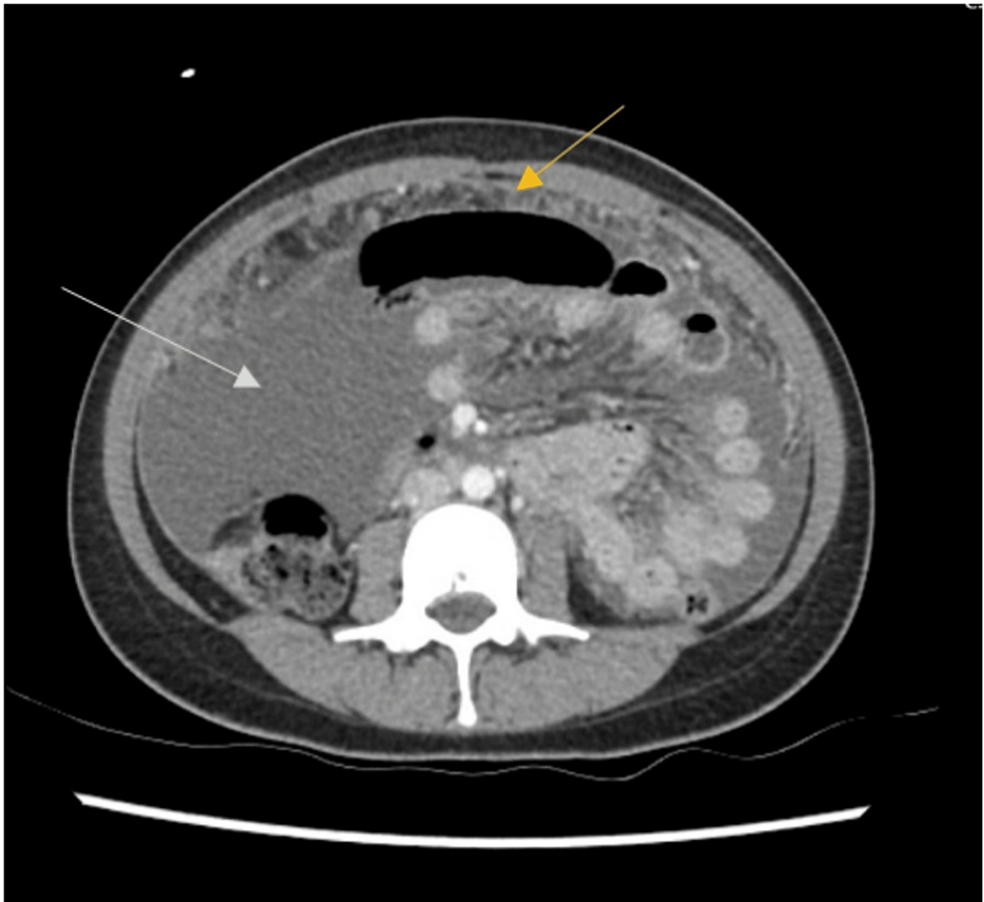
Axial computed tomography scan demonstrating large volume intra-abdominal free fluid (white arrow) with diffuse peritoneal and omental thickening and enhancement (yellow arrow)

**Figure 2:**
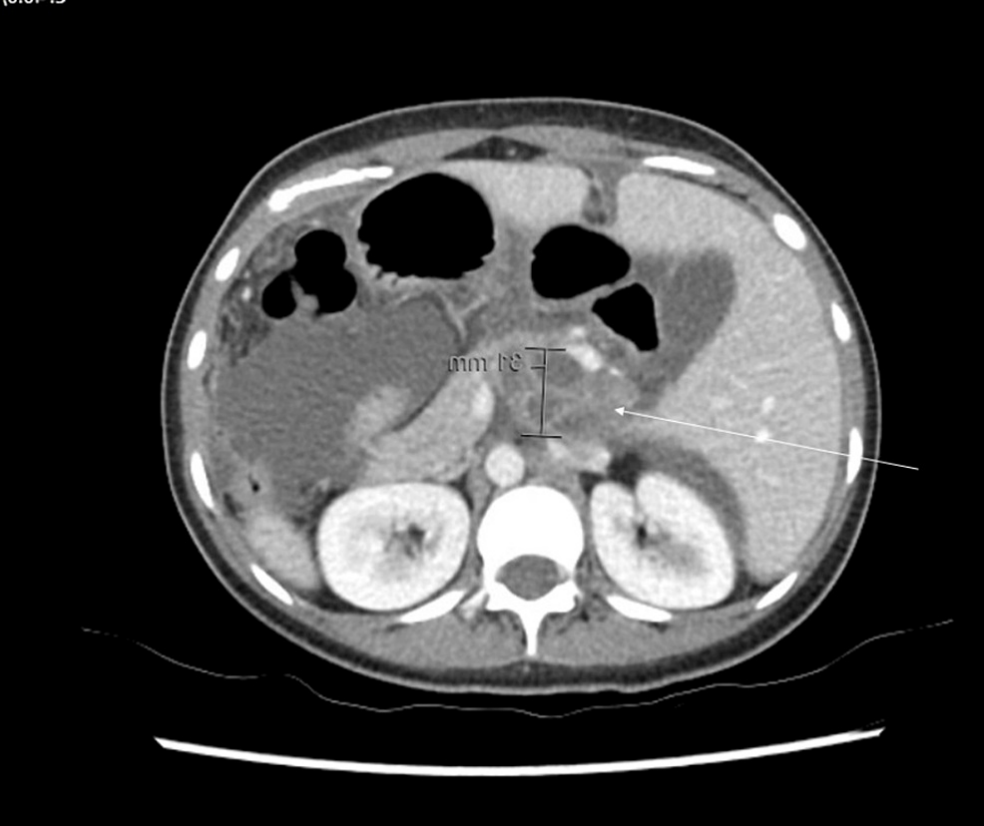
Axial computed tomography image of a multiloculated cystic lesion (3x4.7x5cm) (white arrow) in the upper abdomen between the portal vein and inferior vena cava and between the pancreatic head and caudate lobe of the liver

**Figure 3:**
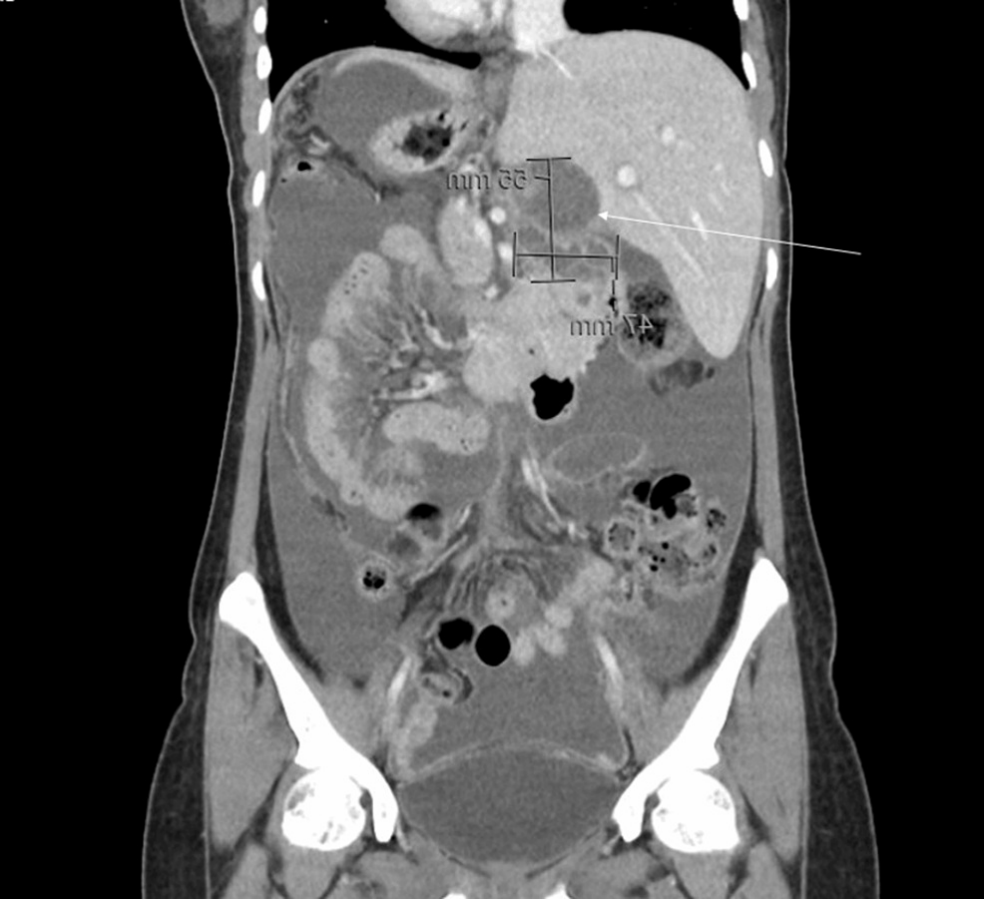
Coronal computed tomography image of the multiloculated cystic lesion (white arrow) as described in Figure 2

Upon examination, she was febrile and had a distended abdomen with generalized tenderness but no peritonism. Blood tests indicated a normal white cell count of 6.7x10^9/L, elevated C-reactive protein at 105, and low albumin at 28g/L. A computed tomography (CT) scan revealed extensive peritoneal disease with significant ascites (Figure 1) as well as the presence of a multiloculated cystic lesion between the portal vein and inferior vena cava and between the pancreatic head and caudate lobe of the liver (Figures 2, 3). Although the differential included benign and malignant pathology, an ascitic fluid confirmed the presence of Mycobacterium tuberculosis. A chest X-ray ruled out thoracic TB and sputum smears were negative. A magnetic resonance imaging scan suggested that the cystic lesion likely represented tuberculous lymphadenopathy.

She was commenced on combination treatment consisting of daily dosing of rifampicin 600mg, isoniazid 300mg, pyrazinamide 1500mg, and ethambutol 800mg, supplemented by pyridoxine 25mg. She was monitored in the hospital until her symptoms improved and she was discharged home on day four to continue TB treatment for the next six months.

## Discussion

Abdominal or peritoneal TB is a rare but significant form of extrapulmonary TB that presents unique challenges in both diagnosis and management. Patients who are immunocompromised with HIV, liver cirrhosis, diabetes, or cancer are at increased risk [2]. Abdominal TB can occur in various parts of the gastrointestinal tract, most commonly in the peritoneum and ileocecal region, typically spreading hematogenously or via ingestion of infected sputum from primary pulmonary TB. However, less than 50% of patients with abdominal TB have pulmonary TB [4].

Abdominal TB presents with non-specific symptoms, making it challenging to differentiate it from other abdominal pathology [2]. Common clinical features include abdominal pain, ascites, weight loss, and fever [5,6]. Additionally, abdominal TB may be misdiagnosed for other peritoneal diseases such as malignancy, inflammatory bowel disease, or spontaneous bacterial peritonitis [5]. Intestinal helminth co-infection is also common in TB patients [7]. The insidious onset of symptoms and the lack of specific signs often contribute to a delay in diagnosis. As such, further diagnostic modalities with imaging studies and biochemical analysis of body fluid are required to confirm the diagnosis. Imaging studies such as abdominal ultrasound and CT may reveal ascites, peritoneal thickening, or omental caking. The gold standard for diagnosing abdominal TB is an analysis of peritoneal fluid or biopsy specimens. Polymerase chain reaction for MTB, adenosine deaminase levels, and cytological examination is diagnostic. Invasive biopsy specimens obtained through laparoscopy or laparotomy can also be used [8].

Once a diagnosis of abdominal TB is confirmed, anti-tuberculous therapy must be initiated as soon as possible. Standard TB treatment regimen involves a combination therapy of daily rifampicin 10-20mg/kg, isoniazid 10-15mg/kg, pyrazinamide 15-30mg/kg, and ethambutol 15-20mg/kg, with treatment duration typically ranging from six to nine months. Pyridoxine 25-50mg is often supplemented at the same time to prevent peripheral neuropathy. In cases of drug-resistant TB, individualized regimens based on drug susceptibility testing may be necessary [9]. Patients are treated with directly observed therapy to ensure adherence and prevent resistance [10].

## Conclusions

In conclusion, peritoneal TB with intestinal helminth co-infection is a rare cause of abdominal pain in countries like Australia with a low endemicity of TB. This case highlights the need for a high index of suspicion for TB when managing patients with unexplained ascites, fevers, and abdominal pain, particularly in those with a history of travel, or previous residence in high-endemic countries. Timely initiation of anti-tuberculous therapy is crucial in symptom improvement, emphasizing the importance of early recognition and treatment to improve outcomes.
